# Comparison of baricitinib, upadacitinib, and tofacitinib mediated regulation of cytokine signaling in human leukocyte subpopulations

**DOI:** 10.1186/s13075-019-1964-1

**Published:** 2019-08-02

**Authors:** Iain B. McInnes, Nicole L. Byers, Richard E. Higgs, Jonathan Lee, William L. Macias, Songqing Na, Robert A. Ortmann, Guilherme Rocha, Terence P. Rooney, Thomas Wehrman, Xin Zhang, Steven H. Zuckerman, Peter C. Taylor

**Affiliations:** 1 Institute of Infection, Immunity and Inflammation, University of Glasgow, University Avenue, Glasgow, G128QQ UK; 2 Eli Lilly and Company, Indianapolis, IN USA; 3 Primity Bio, Fremont, CA USA; 4 Botnar Research Centre, Nuffield Department of Orthopaedics, Rheumatology and Musculoskeletal Sciences, University of Oxford, Oxford, UK

**Keywords:** Baricitinib, Cytokine, Janus kinase, Phosphorylated signal transducer and activator of transcription, Potency, Receptor kinase signaling, Rheumatoid arthritis, Selectivity, Tofacitinib, Upadacitinib

## Abstract

**Background:**

The in vitro pharmacology of baricitinib, upadacitinib, and tofacitinib was evaluated to understand differences among these JAK inhibitors (JAKis) at the cellular level.

**Methods:**

Peripheral blood mononuclear cells from healthy donors were incubated with different JAKis, levels of phosphorylated signal transducer and activator of transcription (pSTAT) were measured following cytokine stimulation, and half maximum inhibitory concentration (IC_50_) values were calculated in phenotypically gated leukocyte subpopulations. Therapeutic dose relevance of the in vitro analysis was assessed using calculated mean concentration-time profiles over 24 h obtained from JAKi-treated subjects. Time above IC_50_ and average daily percent inhibition of pSTAT formation were calculated for each JAKi, cytokine, and cell type.

**Results:**

Distinct JAKis displayed different in vitro pharmacologic profiles. For example, tofacitinib and upadacitinib were the most potent inhibitors of the JAK1/3-dependent cytokines tested (interleukin [IL]-2, IL-4, IL-15, and IL-21) with lower IC_50_ values and increased time above IC_50_ translating to a greater overall inhibition of STAT signaling during the dosing interval. All JAKis tested inhibited JAK1/2-dependent cytokines (e.g., IL-6 and interferon [IFN]-γ), the JAK1/tyrosine kinase 2 (TYK2)-dependent cytokines IL-10 and IFN-α, the JAK2/2-dependent cytokines IL-3 and granulocyte-macrophage colony-stimulating factor (GM-CSF), and the JAK2/TYK2-dependent cytokine granulocyte colony-stimulating factor (G-CSF), but often to significantly differing degrees.

**Conclusions:**

Different JAKis modulated distinct cytokine pathways to varying degrees, and no agent potently or continuously inhibited an individual cytokine signaling pathway throughout the dosing interval. Notably, baricitinib inhibited JAK1/3 signaling to a lesser extent than upadacitinib and tofacitinib, while upadacitinib, baricitinib, and tofacitinib inhibited the signaling of JAK2/2-dependent cytokines, including GM-CSF and IL-3, as well as the signaling of the JAK2/TYK2-dependent cytokine G-CSF.

**Electronic supplementary material:**

The online version of this article (10.1186/s13075-019-1964-1) contains supplementary material, which is available to authorized users.

## Background

The Janus kinase (JAK) family of cytoplasmic protein tyrosine kinases comprises JAK1, JAK2, JAK3, and tyrosine kinase 2 (TYK2). Janus kinases bind to type l and type ll cytokine receptors and transmit extracellular cytokine signals to activate signal transducers and activators of transcription (STATs), which translocate to the nucleus and modulate transcription of effector genes [1]. Recent advances in the treatment of rheumatoid arthritis (RA) have been made with the use of small molecules that inhibit JAKs, specifically targeting cytokine signaling pathways implicated in RA pathogenesis [2–5].

Baricitinib and tofacitinib are JAK inhibitors (JAKis) that have been approved for the treatment of RA, and other JAKis, including upadacitinib, are in clinical development [2]. Baricitinib is approved for the treatment of moderately to severely active RA in adults in over 60 countries including European countries, Japan, and the USA. In vitro kinase assays demonstrate that baricitinib is a selective JAK1 and JAK2 inhibitor with moderate activity against TYK2 and significantly less against JAK3 [6]. Tofacitinib is a potent JAK1 and JAK3 inhibitor but is less active against JAK2 and TYK2 [7]. Upadacitinib is reported as a selective JAK1 inhibitor [8, 9]. Distinct cytokine signaling pathways are mediated by varying JAK complexes, indicating that various JAKis may have differing effects on host inflammatory responses, including those that drive RA.

Herein, we sought to study JAKis that have shown clinical efficacy in the treatment of RA and other autoimmune diseases. The objective of the study was to compare the in vitro cellular pharmacology of baricitinib, upadacitinib, and tofacitinib across relevant leukocyte subpopulations, coupled with their in vivo pharmacokinetics (PK), to determine their effects on distinct cytokine pathways, many implicated in RA pathogenesis.

## Methods

### Leukocyte preparation and experimental design

Whole blood samples from healthy donors (*N* = 4–12) were apheresed, and leukocyte-enriched fractions containing approximately 400 million leukocytes were transferred to the company Primity Bio (Fremont, CA, USA). Immediately following apheresis, approximately 600,000 cells were plated in 100 μL into 96-well plates and incubated with JAKis using a 7-point dose range from 2 to 10,000 nM (four-fold dilutions from the highest concentration) for 1 h prior to stimulation with cytokines for 15 min at 37 °C. Laboratory procedures ensured that compound incubation and cytokine stimulation times were kept constant across cytokines and donors, including when multiple donor samples were being processed. Baricitinib (Eli Lilly and Company), upadacitinib (synthesized by Eli Lilly), and tofacitinib citrate (ApexBio) were prepared as 10-mM stocks in dimethyl sulfoxide. Eight cytokines were used at a concentration of 30 ng/mL (granulocyte colony-stimulating factor [G-CSF], interferon [IFN]-γ, interleukin [IL]-2, IL-4, IL-6, IL-10, IL-15, and IL-21); three others were used at different concentrations: granulocyte-macrophage colony-stimulating factor (GM-CSF) (15 pg/mL), IFN-α (5 ng/mL), and IL-3 (2 ng/mL). For the first eight cytokines, data were generated in two batches; one batch compared baricitinib to tofacitinib (6 donors) and a second batch compared baricitinib to upadacitinib (6 donors). For the remaining cytokines, data were generated in three batches; one batch compared baricitinib to upadacitinib (6 donors) and two other batches compared baricitinib to tofacitinib (2 donors/batch). The choice of cytokine concentrations and incubation conditions were optimized in order to ensure consistent signaling in alternate cell types and phosphorylated STAT (pSTAT) readouts.

### Flow cytometry

After stimulation, cells were fixed, permeabilized, and fluorescence barcoded as previously described [10]. Samples were combined and stained with fluorochrome-conjugated pSTAT1 (Y701), pSTAT3 (Y705), pSTAT5 (Y694), pSTAT6 (Y641), CD3, CD4, CD20, and CD56 antibodies. Multicolor flow cytometry was used to quantify STAT phosphorylation in gated leukocyte subpopulations, and the signals from each sample were de-barcoded for statistical analysis. For a given case (stimulation, cell type, and pSTAT combination), the half maximum inhibitory concentration (IC_50_) was determined if there was a consistent response to the stimulus as described in the “Statistical analysis” section. The primary pSTAT observed for each stimulus is reported. Leukocyte populations were defined as CD20+ (B cells), CD3+CD4+ (CD4+ T cells), CD3+CD4− (CD8+ T cells), CD3−CD56+ (natural killer [NK] cells), and by forward and side scatter (monocytes).

### Statistical analysis

The IC_50_ values for JAKis were determined by analyzing the mean fluorescence intensity (MFI) of cytokine-stimulated samples in the presence of the designated concentration of compound. For a given case (stimulation, cell type, and pSTAT combination), the MFI for unstimulated and stimulated cells was determined for each donor. To ensure that a biologically relevant signal was induced, concentration-response curves (CRCs) were only analyzed when a consistent response to stimulus was observed as described below. Data were analyzed with a statistical model using an integrated data set that included a model term to account for any systematic batch effects.

#### Selection of cases for analysis, fitting, and selection of CRC curves

Two sets of criteria for reporting an IC_50_ value were used: one at the case level and another at the individual curve level. For the CRCs to be estimated for a given case, the case had to satisfy two criteria: (c1) the minimum MFI difference between stimulated and unstimulated reads across all donors was at least 10 fluorescence units and (c2) the *p* value of the one-sided *t* test of the null hypothesis stimulated < unstimulated was at most 0.10. Once a case met these two criteria, four-parameter logistic curves were fit to the 7-point curve concentration data, with the top fixed at the stimulated MFI fluorescence (ensuring all curves for the same donor had the same top parameter). Once fitted, the IC_50_ from a curve was accepted if the following conditions were met: (i1) the *R*^2^ of the fitted curve was above 0.65, (i2) the standard error (SE) of the log(IC_50_) corresponded to a fold change smaller than 8, (i3) the SE of the slope parameter was smaller than 8, and (i4) the difference in the MFI signal between the highest and lowest compound concentrations was larger than both half the distance between the fitted top and bottom parameters for that donor, compound curve, and the stimulated and unstimulated MFI signals for that case. If more than 25% of the curves for a compound were removed, the entire case was removed from further analysis.

#### Estimation and comparison of mean IC_50_ values

Statistical analysis and comparison of IC_50_ values were conducted by fitting statistical mixed effect models to the log(IC_50_), including a random effect for donor and fixed effects for compound for each stimulation, cell type, and pSTAT combination. In cases where data were collected in multiple batches, a batch-fixed effect was added to the model to account for any systematic differences between batches. Reported IC_50_ values corresponded to the least squares means of the compound effects from the model. Reported *p* values were adjusted for multiplicity by using a Bonferroni correction within each stimulant and pSTAT combination.

#### Pharmacokinetic profiles

The PK profiles of baricitinib were estimated from a two-compartment model with zero-order absorption that was developed using data from patients with RA from phase 2 and phase 3 clinical trials with once daily (QD) 2- or 4-mg doses [11]. The PK profiles of upadacitinib were obtained from published data using RA scaled healthy volunteer profiles in a dose-proportional manner for QD 15- or 30-mg doses [12, 13]. The PK profiles of tofacitinib were estimated from a one-compartment model with zero-order absorption using data from patients with RA from phase 3 clinical trials with twice daily 5- or 10-mg doses [14].

#### Estimation and comparison of time above IC_50_ and daily percent inhibition

The individual 4PL CRCs were combined with population PK curves to calculate time above IC_50_ and average daily percent inhibition. Protein binding effects were accounted for by replacing the in vitro IC_50_ with an adjusted IC_50_ value computed by dividing the IC_50_ value for each donor by the proportion of compound unbound (baricitinib 50%, upadacitinib 54%, and tofacitinib 60%). The time above IC_50_ was defined as the time the (linearly interpolated) PK concentration was above the adjusted IC_50_. Protein-bound adjusted CRCs were constructed by replacing the in vitro IC_50_ value with the adjusted value. The average daily percent inhibition for a subject was obtained by entering the steady-state PK concentrations into the adjusted CRCs, computing the area under this curve, and dividing it by 24 h. Using the individual donor values for time above IC_50_ and average daily percent inhibition, the same mixed model used to fit the log(IC_50_) values was used to estimate and compare mean times above IC_50_ and percent inhibitions. No transformations were undertaken to keep the estimates within the 0–24-h range (for time above IC_50_) and 0–100% (for average daily inhibition). The values were truncated in a few cases where the estimates fell outside the range.

## Results

### IC_50_ values for JAKis in cytokine-stimulated human PBMC preparations

We first determined the specificity and potency of different JAKis in inhibiting cytokine-induced pSTATs in human peripheral blood mononuclear cells (PBMCs). Following cytokine stimulation, quantification of the inhibition of pSTATs elucidated that IC_50_ values for any given JAKi were similar across cell types and were dose-dependent. However, differences in IC_50_ values were observed between the JAKis assessed. For the JAK1/3-dependent cytokines IL-2, IL-4, IL-15, and IL-21, IC_50_ values were lowest (most potent) for upadacitinib and tofacitinib and highest (least potent) for baricitinib (Table 1 and Additional file 1: Table S1).

**Table 1:** IC_50_ values in CD4+ T cells, NK cells, and monocytes

Stimulation/pSTAT	CD4+ T cells			NK cells			Monocytes		
	Bari (nM)	Upa (nM)	Tofa (nM)	Bari (nM)	Upa (nM)	Tofa (nM)	Bari (nM)	Upa (nM)	Tofa (nM)
JAK1/3-dependent cytokines									
IL-2/pSTAT5	29	10**	11***	44	27*	15***	NS		
IL-4/pSTAT6	48	18***	18***	22	8*	8***	45	22**	35*
IL-15/pSTAT5	40	17***	15***	67	40*	22***	NS		
IL-21/pSTAT3	64	20***	22***	62	24**	21***	85	34	37
JAK2/2- or JAK2/TYK2-dependent cytokines									
IL-3/pSTAT5	NS			NS			26	12*	102***
G-CSF/pSTAT3	NS			NS			65	84	97**
GM-CSF/pSTAT5	NS			NS			30	13***	97***
JAK1/JAK2/TYK2-dependent cytokines									
IL-6/pSTAT3	61	58	56	NS			48	43	40
IL-10/pSTAT3	68	87	55	87	124	74	142	80***	104*
IFN-γ/pSTAT1	NS			NS			38	30	46***
IFN-α/pSTAT1	64	40	132***	76	69	121***	97	44**	163***
IFN-α/pSTAT3	27	17	51***	NS			14	6*	23***
IFN-α/pSTAT5	23	14	36**	NS			13	5**	22***

Reported IC_50_ values are least squares estimates of mixed models as described in the “Statistical analysis” section. For G-CSF, IFN-γ, IL-2, IL-4, IL-6, IL-10, IL-15, and IL-21, reported IC_50_ values are based on two batches of data amounting to 12 donors for baricitinib and 6 donors for upadacitinib and tofacitinib; for GM-CSF, IFN-α, and IL-3, reported IC_50_ values are based on three batches of data amounting to 10 donors for baricitinib, 6 donors for upadacitinib, and 4 donors for tofacitinib. The primary pSTAT observed for each stimulus is reported in the table. Protein binding was not accounted for in the IC_50_ calculations

*p < 0.01, **p < 0.001, ***p < 0.0001 compared to baricitinib

*Bari* baricitinib, *G-CSF* granulocyte colony-stimulating factor, *GM-CSF* granulocyte-macrophage colony-stimulating factor, *IC*_*50*_ half maximum inhibitory concentration, *IFN* interferon, *IL* interleukin, *JAK* Janus kinase, *NK* natural killer, *NS* no stimulation, *pSTAT* phosphorylated signal transducer and activator of transcription, *Tofa* tofacitinib, *TYK* tyrosine kinase, *Upa* upadacitinib

Signal inhibition was assessed in monocytes for the JAK2/2-dependent cytokines IL-3 and GM-CSF and the JAK2/TYK2-dependent cytokine G-CSF (Table 1). IC_50_ values for IL-3, GM-CSF, and G-CSF signal inhibition indicated highest potency for upadacitinib and baricitinib and reduced potency for tofacitinib (Table 1). A similar pattern was observed for IL-3 signal inhibition in B cells (Additional file 1: Table S1).

IC_50_ values for IL-6 (JAK1/2) signaling in CD4+ T cells and monocytes, and for IL-10- (JAK1/TYK2) stimulated T cells and NK cells, were similar for baricitinib, upadacitinib, and tofacitinib (Table 1 and Additional file 1: Table S1). IC_50_ values for pSTAT3 inhibition in IL-10-stimulated monocytes indicated greatest potency for upadacitinib and tofacitinib and reduced potency for baricitinib (Table 1). IC_50_ values for pSTAT1 inhibition in IFN-γ- (JAK1/2) stimulated monocytes indicated highest potency for baricitinib and upadacitinib and reduced potency for tofacitinib (Table 1). IC_50_ values for pSTAT1 inhibition in IFN-γ-stimulated B cells were similar for baricitinib, upadacitinib, and tofacitinib (Additional file 1: Table S1). Across cell types that had a response, IC_50_ values for IFN-α- (JAK1/TYK2) induced pSTAT1, 3, and 5 indicated the highest potency for upadacitinib and baricitinib and lower potency for tofacitinib (Table 1 and Additional file 1: Table S1).

### Time above IC_50_

**Fig. 1:**
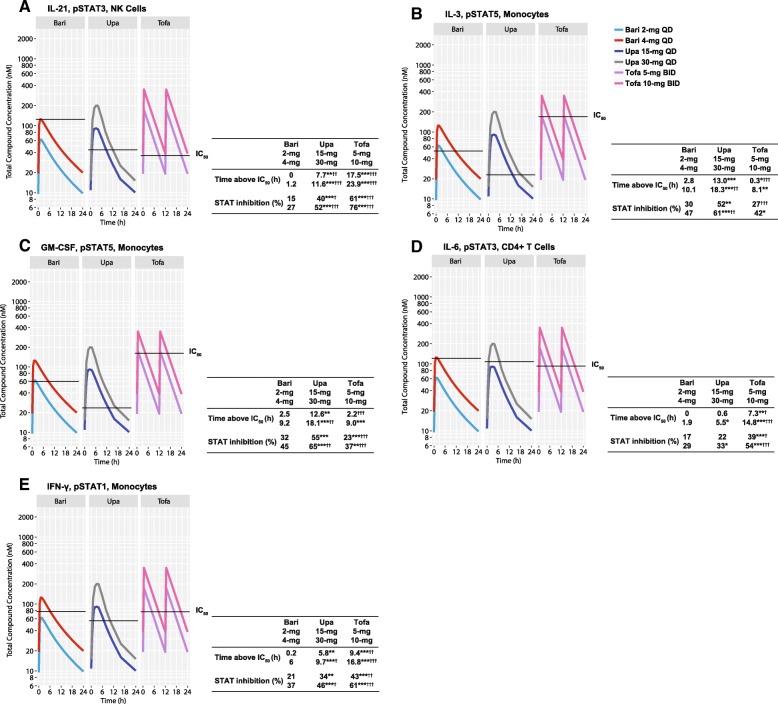
JAKi exposure curves for select cytokines. The number of hours per day JAKi concentrations are above IC_50_ values is shown for IL-21/pSTAT3 in NK cells (**a**), IL-3/pSTAT5 in monocytes (**b**), GM-CSF/pSTAT5 in monocytes (**c**), IL-6/pSTAT3 in CD4+ T cells (**d**), and IFN-γ/pSTAT1 in monocytes (**e**). Panels **a**–**e** include the average daily percent STAT inhibition. Protein binding was accounted for in the calculation of hours per day above IC_50_ and average daily percent STAT inhibition, and the IC_50_ values were corrected for the compound-bound proportion. *p < 0.01, **p < 0.001, ***p < 0.0001 compared to baricitinib 2 mg; ^†^*p* < 0.01, ^††^*p* < 0.001, ^†††^*p* < 0.0001 compared to baricitinib 4 mg. Bari, baricitinib; BID, twice daily; GM-CSF, granulocyte-macrophage colony-stimulating factor; h, hours; IC_50_, half maximum inhibitory concentration; IFN, interferon; IL, interleukin; JAK, Janus kinase; JAKi, JAK inhibitor; NK, natural killer; pSTAT, phosphorylated STAT; QD, once daily; STAT, signal transducer and activator of transcription; Tofa, tofacitinib; Upa, upadacitinib

The potential clinical relevance of the foregoing was next evaluated by examining the likely cell subset-specific inhibition pattern in patients over a 24-h period, according to the reported exposure levels (plasma concentrations during the daily dosing cycle) for each agent. This analysis captures “time-adjusted” inhibitory activity in terms of immune modulatory function and could differ significantly among agents with distinct PK properties. The number of hours per day JAKi concentrations were above IC_50_ values for each cytokine/pSTAT combination was determined using calculated IC_50_ values and exposure data from JAKi-treated subjects. For the JAK1/3-dependent cytokines IL-2, IL-4, IL-15, and IL-21, tofacitinib spent the most time per day above IC_50_ for the pSTAT induced by each cytokine across cell types tested; time above IC_50_ was generally sequentially less for upadacitinib and baricitinib (Table 2 and Additional file 1: Tables S2–S4, and Fig. 1a). To illustrate this pattern in NK cells, for IL-15/pSTAT5, time above IC_50_ was 17 and 23 h for tofacitinib 5- and 10-mg doses, 3.4 and 8.1 h for upadacitinib 15- and 30-mg doses, and 0 and 1 h for baricitinib 2- and 4-mg doses, respectively. A similar relationship was observed for IL-21/pSTAT3 (Table 2 and Additional file 1: Tables S2–S4, and Fig. 1a).

**Table 2:** Hours per day above IC_50_ in CD4+ T cells, NK cells, and monocytes: baricitinib 4 mg

Stimulation/pSTAT	CD4+ T cells			NK cells			Monocytes		
	Bari 4 mg	Upa 15 mg 30 mg	Tofa 5 mg 10 mg	Bari 4 mg	Upa 15 mg 30 mg	Tofa 5 mg 10 mg	Bari 4 mg	Upa 15 mg 30 mg	Tofa 5 mg 10 mg
JAK1/3-dependent cytokines									
IL-2/pSTAT5	9	15.3* 20.6**	23.8*** 24.2***	4.5	6.6 10.5**	21.4*** 24.0***	NS		
IL-4/pSTAT6	3.7	9.9 14.4**	19.6*** 24.2***	12.6	17.5 22.3*	24.0*** 24.0***	4.5	8.2 12.3**	12.3*** 19.7***
IL-15/pSTAT5	5.5	10.4** 14.5***	21.2*** 24.0***	1	3.4 8.1**	17.0*** 23.0***	NS		
IL-21/pSTAT3	0.7	9.0*** 13.1***	17.0*** 23.6***	1.2	7.7** 11.6***	17.5*** 23.9***	0	4.4 8.6*	12.5* 18.9**
JAK2/2- or JAK2/TYK2-dependent cytokines									
IL-3/pSTAT5	NS			NS			10.1	13.0 18.3**	0.3*** 8.1
G-CSF/pSTAT3	NS			NS			1.4	0.1 3.2	2.3 8.5*
GM-CSF/pSTAT5	NS			NS			9.2	12.6 18.1**	2.2*** 9.0
JAK1/JAK2/TYK2-dependent cytokines									
IL-6/pSTAT3	1.9	0.6 5.5	7.3* 14.8***	NS			3.6	3.1 7.6*	10.8* 18.2***
IL-10/pSTAT3	1.2	0 2.8	7.6* 15.1***	0.4	0 0.5	4.2* 11.8***	0	0.6 3.5	0.8 7.8***
IFN-γ/pSTAT1	NS			NS			6	5.8 9.7*	9.4** 16.8***
IFN-α/pSTAT1	0.9	3.6 8.1**	0 5.2	0.2	0 4.8**	0.1 5.7***	0	2.9 7.5***	0 2.8***
IFN-α/pSTAT3	9.6	10.4 15.0	8.0 15.5***	NS			18.2	21.7 23.7	16.6 23.2*
IFN-α/pSTAT5	12	12.9 17.2	12.2 19.6***	NS			19.8	21.9 23.5	17.2* 23.9**

Reported hours per day above IC_50_ are least squares estimates of mixed models as described in the “Statistical analysis” section. Protein binding was accounted for in the calculations. JAKis were administered once daily (baricitinib and upadacitinib) or twice daily (tofacitinib)

*p < 0.01, **p < 0.001, ***p < 0.0001 compared to baricitinib 4 mg

*Bari* baricitinib, *G-CSF* granulocyte colony-stimulating factor, *GM-CSF* granulocyte-macrophage colony-stimulating factor, *IC*_*50*_ half maximum inhibitory concentration, *IFN* interferon, *IL* interleukin, *JAK* Janus kinase, *JAKi* JAK inhibitor, *NK* natural killer, *NS* no stimulation, *pSTAT* phosphorylated signal transducer and activator of transcription, *Tofa* tofacitinib, *TYK* tyrosine kinase, *Upa* upadacitinib

For IL-3 and GM-CSF (JAK2/2), time above IC_50_ for pSTAT5 inhibition in monocytes was generally highest for upadacitinib and lower for baricitinib and tofacitinib (Table 2 and Additional file 1: Table S3, and Fig. 1b, c). To illustrate this pattern, for GM-CSF/pSTAT5 in monocytes, time above IC_50_ was 12.6 and 18.1 h for upadacitinib 15- and 30-mg doses, 2.5 and 9.2 h for baricitinib 2- and 4-mg doses, and 2.2 and 9.0 h for tofacitinib 5- and 10-mg doses, respectively (Table 2 and Additional file 1: Table S3, and Fig. 1c). For G-CSF (JAK2/TYK2) in monocytes, time spent above IC_50_ for pSTAT3 inhibition was most for tofacitinib and upadacitinib and least for baricitinib (Table 2 and Additional file 1: Table S3).

For IL-6-induced CD4+ T cells and monocytes, time above IC_50_ for pSTAT3 inhibition was generally highest for tofacitinib, decreased for upadacitinib, and least for baricitinib (Table 2 and Additional file 1: Table S3, and Fig. 1d). To illustrate this pattern, for IL-6/pSTAT3 in CD4+ T cells, time above IC_50_ was 7.3 and 14.8 h for tofacitinib 5- and 10-mg doses, 0.6 and 5.5 h for upadacitinib 15- and 30-mg doses, and 0 and 1.9 h for baricitinib 2- and 4-mg doses, respectively (Table 2 and Additional file 1: Table S3, and Fig. 1d). This pattern was also generally observed for JAKis in IFN-γ-induced cell types (Table 2 and Additional file 1: Tables S2–S4, and Fig. 1e). For cell types stimulated by IL-10, time above IC_50_ for pSTAT3 inhibition was generally highest for tofacitinib and least for upadacitinib and baricitinib (Table 2 and Additional file 1: Tables S2–S4). For cell types stimulated by IFN-α, time above IC_50_ for pSTAT inhibition was generally the most for upadacitinib and tofacitinib and the least for baricitinib (Table 2 and Additional file 1: Table S2–S4).

### Percent STAT inhibition

The JAK inhibitors were next compared by calculating the percent inhibition of STAT phosphorylation over a 24-h interval to determine the functional consequence of JAK inhibition reflected by the magnitude and duration of inhibition of the phosphorylation of their most proximal substrates. Concentration-response curves combined with exposure data for JAKi-treated subjects revealed cytokine-dependent similarities and differences in the magnitude of pSTAT inhibition (Table 3 and Additional file 1: Tables S5–S7). Percent STAT inhibition for IL-2, IL-4, IL-15, and IL-21 signaling (JAK1/3) was generally highest for tofacitinib and upadacitinib and least for baricitinib (Table 3 and Additional file 1: Tables S5–S7, and Fig. 1a). For example, in NK cells, percent STAT inhibition for IL-15/pSTAT5 was 62% and 79% for tofacitinib 5- and 10-mg doses, 27% and 39% for upadacitinib 15- and 30-mg doses, and 12% and 24% for baricitinib 2- and 4-mg doses, respectively. A similar relationship was observed for IL-21/pSTAT3 (Table 3 and Additional file 1: Table S6, and Fig. 1a).

**Table 3:** Average daily percent STAT inhibition in CD4+ T cells, NK cells, and monocytes: baricitinib 4 mg

Stimulation/pSTAT	CD4+ T cells			NK cells			Monocytes		
	Bari 4 mg	Upa 15 mg 30 mg	Tofa 5 mg 10 mg	Bari 4 mg	Upa 15 mg 30 mg	Tofa 5 mg 10 mg	Bari 4 mg	Upa 15 mg 30 mg	Tofa 5 mg 10 mg
JAK1/3-dependent cytokines									
IL-2/pSTAT5	44	60* 71***	78*** 89***	34	36 48**	72*** 85***	NS		
IL-4/pSTAT6	30	45* 57***	69*** 84***	52	61 71**	82*** 91***	33	40 52**	51*** 70***
IL-15/pSTAT5	36	47** 59***	72*** 85***	24	27 39**	62*** 79***	NS		
IL-21/pSTAT3	27	44*** 55***	61*** 76***	27	40* 52***	61*** 76***	22	34 45*	46* 63**
JAK2/2- or JAK2/TYK2-dependent cytokines									
IL-3/pSTAT5	NS			NS			47	52 61**	27*** 42
G-CSF/pSTAT3	NS			NS			25	17* 26	27 40**
GM-CSF/pSTAT5	NS			NS			45	55 65**	23*** 37***
JAK1/JAK2/TYK2-dependent cytokines									
IL-6/pSTAT3	29	22 33	39* 54***	NS			32	27 39	46*** 61***
IL-10/pSTAT3	27	21 29	39* 53***	23	14* 21	34** 48***	14	17 26*	25*** 40***
IFN-γ/pSTAT1	NS			NS			37	34 46*	43** 61***
IFN-α/pSTAT1	29	32 43*	24** 36**	25	21 31	23 37***	21	28 40***	21 32***
IFN-α/pSTAT3	45	48 61	40 55*	NS			62	72 81*	60 76*
IFN-α/pSTAT5	50	52 64	49 64**	NS			67	75 83*	62* 78**

Reported average daily percent STAT inhibition values are least squares estimates of mixed models as described in the “Statistical analysis” section. Protein binding was accounted for in the calculations. JAKis were administered once daily (baricitinib and upadacitinib) or twice daily (tofacitinib)

*p < 0.01, **p < 0.001, ***p < 0.0001 compared to baricitinib 4 mg

*Bari* baricitinib, *G-CSF* granulocyte colony-stimulating factor, *GM-CSF* granulocyte-macrophage colony-stimulating factor, *IFN* interferon, *IL* interleukin, *JAK* Janus kinase, *JAKi* JAK inhibitor, *NK* natural killer, *NS* no stimulation, *pSTAT* phosphorylated STAT, *STAT* signal transducer and activator of transcription, *Tofa* tofacitinib, *TYK* tyrosine kinase, *Upa* upadacitinib

Percent STAT inhibition for IL-3 and GM-CSF (JAK2/2) in monocytes was generally highest for upadacitinib and sequentially less so for baricitinib and tofacitinib (Table 3 and Additional file 1: Table S6, and Fig. 1b, c). Percent STAT inhibition for GM-CSF/pSTAT5 in monocytes was 55% and 65% for upadacitinib 15- and 30-mg doses, 32% and 45% for baricitinib 2- and 4-mg doses, and 23% and 37% for tofacitinib 5- and 10-mg doses, respectively (Table 3 and Additional file 1: Table S6, and Fig. 1c). A similar trend was observed for IL-3-induced B cells. Percent STAT inhibition for G-CSF/pSTAT3 (JAK2/TYK2) in monocytes was highest for tofacitinib and less for upadacitinib and baricitinib (Table 3 and Additional file 1: Table S6).

For cells stimulated by IL-6 (JAK1/2) and IL-10 (JAK1/TYK2), percent STAT inhibition was generally highest for tofacitinib and less for upadacitinib and baricitinib (Table 3 and Additional file 1: Table S5–S7, and Fig. 1d). For IL-6/pSTAT3 in CD4+ T cells, percent STAT inhibition was 39% and 54% for tofacitinib 5- and 10-mg doses, 22% and 33% for upadacitinib 15- and 30-mg doses, and 17% and 29% for baricitinib 2- and 4-mg doses, respectively (Table 3 and Additional file 1: Table S6, and Fig. 1d). A similar trend was observed in IFN-γ- (JAK1/2) induced cell types (Table 3 and Additional file 1: Tables S5–S7, and Fig. 1e). The percent STAT inhibition for IFN-α- (JAK1/TK2) induced pSTAT1, 3, and 5 was generally highest for upadacitinib and tofacitinib and less for baricitinib, across cell types (Table 3 and Additional file 1: Table S5).

## Discussion

The introduction of multiple JAKis into clinical practice, each with distinct selectivity across the JAK family members as determined by in vitro kinase assays, poses obvious questions as to the relative functional impact of such differences. Herein, we characterized the in vitro cellular pharmacology of baricitinib, upadacitinib, and tofacitinib, coupled to their in vivo PK, to determine their effects (at human oral doses that are approved or included in late phase clinical studies) on distinct cytokine pathways involved in the pathogenesis of RA.

Tofacitinib and upadacitinib, for example, were the most potent inhibitors of the JAK1/3-dependent cytokines tested. Moreover, lower IC_50_ values and increased time above IC_50_ for tofacitinib and upadacitinib, compared for example with baricitinib, translated to a greater overall inhibition of STAT signaling during the 24-h dosing interval for JAK1/3-dependent cytokines. Inhibition of this particular pathway may have potential impact on lymphocyte activation. Notably, IL-15 and IL-21 induce the maturation and function of NK cells and this may be of relevance to the changes in peripheral blood NK cell numbers reported in JAKi trials in RA [9, 15–19]. Relationships between the durability of STAT inhibition by any given JAKi and overall changes in lymphocyte subpopulations observed in RA clinical trials with these molecules remains to be determined.

Upadacitinib, baricitinib, and tofacitinib inhibited the JAK2/2 or JAK2/TYK2 signaling cytokines IL-3, GM-CSF, and G-CSF, albeit to varying degrees. Upadacitinib proved the most potent inhibitor of IL-3 and GM-CSF (JAK2/2), followed by baricitinib and tofacitinib, while tofacitinib proved the most potent inhibitor of G-CSF (JAK2/TYK2), followed by upadacitinib and baricitinib. Both G-CSF and GM-CSF may contribute to RA pathogenesis through the activation, differentiation, and survival of myeloid cells [20]. Indeed, blockade of GM-CSF receptor with the humanized monoclonal antibody mavrilimumab was effective in a phase 2 clinical trial in RA [21]. Baricitinib and other JAKis with activity against JAK2 may attenuate RA pathogenesis in part through inhibiting GM-CSF-mediated cellular responses.

With respect to JAK1/2-dependent cytokine signaling, baricitinib, upadacitinib, and tofacitinib were all inhibitors of IL-6 and IFN-γ (JAK1/2), and IL-10 and IFN-α (JAK1/TYK2) signaling, although differences in potency emerged. Tofacitinib proved the most potent inhibitor of IL-6, IFN-γ, and IL-10 signaling, followed by upadacitinib and baricitinib, while upadacitinib and tofacitinib were the most potent inhibitors of IFN-α signaling. Interleukin-6 is a pleiotropic pro-inflammatory cytokine that contributes to synovial inflammation, articular joint destruction, and some of the systemic features observed in RA [22]. Baricitinib and other JAKis may be effective in the treatment of RA in part by IL-6 inhibition, which has been validated as a therapeutic target in RA patients by the monoclonal antibody tocilizumab [23]. Interleukin-10, IFN-α, and IFN-γ are also likely contributors to RA pathogenesis, and inhibition of these responses may contribute to the mechanism of JAKis in RA treatment; lesser inhibition of IL-10 may be desirable as the net effects of this cytokine have been described as anti-inflammatory in RA [4, 24].

Upadacitinib has been reported to be a selective JAK1 inhibitor [8, 9]. Data in this study, however, showed that at clinically relevant doses, upadacitinib was the most potent inhibitor among the drugs tested of the JAK2-dependent cytokines IL-3 and GM-CSF. These findings indicate that upadacitinib would also inhibit cytokines other than those primarily dependent upon JAK1 at clinically effective concentrations. Data in this study also demonstrate that tofacitinib has moderate activity against JAK2 and TYK2, in addition to activity against JAK1 and JAK3. Together, these findings suggest that conclusions about potency and selectivity drawn from refined in vitro kinase assays may differ when considering the more biologically relevant concept of signal blockade at the cellular level in the context of circulating drug concentrations at doses used in humans.

There are limitations to the conclusions that can be drawn from this analysis. One potential limitation with this study is that high concentrations of cytokine can right shift the IC_50_ values if the STAT substrate is limiting. This could potentially be limiting our interpretation of the data for upadacitinib. However, we investigated this phenomenon and adjusted the cytokine concentrations where necessary, but some cytokines were either insensitive to concentration or could not be lowered without compromising the data in an alternate cell type or STAT readout. It would have been valuable to assess the ability of each JAKi to impair the half maximal response concentration (EC_50_) of these cytokines, but would require extensive tuning of each cytokine in each cell type, which is well beyond the scope of this initial report. Another limitation was that the statistical analysis of this study used a single, average PK profile and did not reflect inter-subject variability. Furthermore, all IC_50_ values were calculated from PBMCs derived from healthy volunteers and extrapolated where available to RA patient exposure curves. Finally, while this study describes a reproducible cellular test system in which to test the molecules, the drugs were introduced to unstimulated healthy volunteer cells, which were then stimulated. Looking at how such molecules perform in previously activated cells may be more relevant to in vivo inflammatory disease conditions. This may warrant future study, for instance using samples from patients with active inflammatory disease.

## Conclusions

These data demonstrate that JAKis display different in vitro pharmacologic profiles which, when coupled with their in vivo PK profiles, suggest that they may work via modulating differing cytokine pathways to varying degrees and durations over a 24-h dosing interval. None of the JAKis studied completely or continuously inhibited an individual cytokine signaling pathway over the dosing interval when assessed by STAT inhibition or time over IC_50_, respectively. These observations may have implications for the efficacy and safety profiles observed with different JAKis across different disease states. This in turn may inform JAKi development tailored to capitalize on the most clinically beneficial pharmacological features that will emerge as several agents are established in clinical practice.

## Additional file

Additional file 1: **Table S1.** IC_50_ values in B cells and CD8+ T cells. **Table S2.** Hours per day above IC_50_ in B cells and CD8+ T cells: baricitinib 4-mg. **Table S3.** Hours per day above IC_50_ in CD4+ T cells, NK cells, and monocytes: baricitinib 2-mg. **Table S4.** Hours per day above IC_50_ in B cells and CD8+ T cells: baricitinib 2-mg. **Table S5.** Average daily percent STAT inhibition in B cells and CD8+ T cells: baricitinib 4-mg. **Table S6.** Average daily percent STAT inhibition in CD4+ T cells, NK cells, and monocytes: baricitinib 2-mg. **Table S7.** Average daily percent STAT inhibition in B cells and CD8+ T cells: baricitinib 2-mg. **Figure S1.** Select representative histogram plots for cytokine-induced STAT phosphorylation (DOCX 278 kb)

## Data Availability

Eli Lilly and Company provides access to relevant anonymized patient-level data from studies on approved medicines and indications as defined by the sponsor-specific information on www.clinicalstudydatarequest.com. For details on submitting a request, see the instructions provided at www.clinicalstudydatarequest.com.

## Abbreviations

CRC: Concentration-response curve
EC^50^: Half maximal response concentration
CRC: Concentration-response curve
G-CSF: Granulocyte colony-stimulating factor
GM-CSF: Granulocyte-macrophage colony-stimulating factor
IC_50_: Half maximum inhibitory concentration
IFN: Interferon
IL: Interleukin
JAK: Janus kinase
JAKi: JAK inhibitor
MFI: Mean fluorescence intensity
NK: Natural killer
PBMC: Peripheral blood mononuclear cell
PK: Pharmacokinetic
pSTAT: Phosphorylated STAT
QD: Once daily
RA: Rheumatoid arthritis
SE: Standard error
STAT: Signal transducer and activator of transcription
TYK: Tyrosine kinase
