# Supplementary material for: Comparison of baricitinib, upadacitinib, and tofacitinib mediated regulation of cytokine signaling in human leukocyte subpopulations
Source: Arthritis Res Ther. 2019 Aug 2;21:183. doi: 10.1186/s13075-019-1964-1 (PMC6679539; doi:10.1186/s13075-019-1964-1)
Supplement: Supplementary file 1 — Table S1. IC50 values in B cells and CD8+ T cells. Table S2. Hours per day above IC50 in B cells and CD8+ T cells: baricitinib 4-mg. Table S3. Hours per day above IC50 in CD4+ T cells, NK cells, and monocytes: baricitinib 2-mg. Table S4. Hours per day above IC50 in B cells and CD8+ T cells: baricitinib 2-mg. Table S5. Average daily percent STAT inhibition in B cells and CD8+ T cells: baricitinib 4-mg. Table S6. Average daily percent STAT inhibition in CD4+ T cells, NK cells, and monocytes: baricitinib 2-mg. Table S7. Average daily percent STAT inhibition in B cells and CD8+ T cells: baricitinib 2-mg. Figure S1. Select representative histogram plots for cytokine-induced STAT phosphorylation (DOCX 278 kb) [file 13075_2019_1964_MOESM1_ESM.docx]

**Additional file 1**

**Table S1. IC_50_ values in B cells and CD8+ T cells^a^**

|  | **B cells** | | | **CD8+ T cells** | | |
| --- | --- | --- | --- | --- | --- | --- |
| **Stimulation/**  **pSTAT** | Bari  (nM) | Upa  (nM) | Tofa  (nM) | Bari  (nM) | Upa  (nM) | Tofa  (nM) |
| **JAK1/3-dependent cytokines** |  |  |  |  |  |  |
| IL-2/pSTAT5 | NS | | | 27 | 14* | 10*** |
| IL-4/pSTAT6 | 122 | 42*** | 55** | 30 | 14 | 11*** |
| IL-15/pSTAT5 | NS | | | 65 | 32* | 24*** |
| IL-21/pSTAT3 | 87 | 25** | 28*** | 68 | 24*** | 23*** |
| **JAK2/2- or JAK2/TYK2- dependent cytokines** |  |  |  |  |  |  |
| IL-3/pSTAT5 | 85 | 92 | 346*** | NS | | |
| G-CSF/pSTAT3 | NS | | | NS | | |
| GM-CSF/pSTAT5 | NS | | | NS | | |
| **JAK1/JAK2/TYK2-dependent cytokines** |  |  |  |  |  |  |
| IL-6/pSTAT3 | NS | | | NS | | |
| IL-10/pSTAT3 | NS | | | 78 | 105 | 70 |
| IFN-γ/pSTAT1 | 24 | 23 | 30 | NS | | |
| IFN-α/pSTAT1 | 100 | 80 | 187*** | 79 | 57 | 159*** |
| IFN-α/pSTAT3 | NS | | | NS | | |
| IFN-α/pSTAT5 | NS | | | NS | | |

^a^Reported IC_50_ values are least squares estimates of mixed models as described in the “Statistical analysis” section. For G-CSF, IFN-γ, IL-2, IL-4, IL-6, IL-10, IL-15, and IL-21, reported IC_50_ values are based on two batches of data amounting to 12 donors for baricitinib, and 6 donors for upadacitinib and tofacitinib; for GM-CSF, IFN-α, and IL-3, reported IC_50_ values are based on three batches of data amounting to 10 donors for baricitinib, 6 donors for upadacitinib, and 4 donors for tofacitinib. The primary pSTAT observed for each stimulus is reported in the table. Protein binding was not accounted for in the IC_50_ calculations.

*p<0.01, **p<0.001, ***p<0.0001 compared to baricitinib.

bari, baricitinib; G-CSF, granulocyte colony-stimulating factor; GM-CSF, granulocyte-macrophage colony-stimulating factor; IC_50_, half maximum inhibitory concentration; IFN, interferon; IL, interleukin; JAK, Janus kinase; NS, no stimulation; pSTAT, phosphorylated signal transducer and activator of transcription; tofa, tofacitinib; TYK, tyrosine kinase; upa, upadacitinib.

**Table S2. Hours per day above IC_50_ in B cells and CD8+ T cells: baricitinib 4-mg^a,b^**

|  | **B cells** | | | **CD8+ T cells** | | |
| --- | --- | --- | --- | --- | --- | --- |
| **Stimulation/**  **pSTAT** | Bari  4-mg | Upa  15-mg  30-mg | Tofa  5-mg  10-mg | Bari  4-mg | Upa  15-mg  30-mg | Tofa  5-mg  10-mg |
| **JAK1/3-dependent cytokines** |  |  |  |  |  |  |
| IL-2/pSTAT5 | NS | | | 10.5 | 12.4  17.0** | 22.0**  23.2*** |
| IL-4/pSTAT6 | 0 | 3.3  7.8** | 7.3*  14.8*** | 8.8 | 12.1  16.7* | 23.5***  24.1*** |
| IL-15/pSTAT5 | NS | | | 0.9 | 5.7*  9.6*** | 16.2***  23.1*** |
| IL-21/pSTAT3 | 0.1 | 7.4***  11.3*** | 14.4***  21.8*** | 0.8 | 7.7***  11.7*** | 16.7***  23.3*** |
| **JAK2/2- or JAK2/TYK2-dependent cytokines** |  |  |  |  |  |  |
| IL-3/pSTAT5 | NS | | | NS | | |
| G-CSF/pSTAT3 | NS | | | NS | | |
| GM-CSF/pSTAT5 | NS | | | NS | | |
| **JAK1/JAK2/TYK2-dependent cytokines** |  |  |  |  |  |  |
| IL-6/pSTAT3 | NS | | | NS | | |
| IL-10/pSTAT3 | NS | | | 0.3 | 0  1.5 | 4.6*  12.3*** |
| IFN-γ/pSTAT1 | 11.1 | 7.9  12.5 | 14.1  21.1*** | NS | | |
| IFN-α/pSTAT1 | 0 | 0  3.9*** | 0  1.3 | 0.2 | 0.7  5.7 | 0.1  3.3* |
| IFN-α/pSTAT3 | NS | | | NS | | |
| IFN-α/pSTAT5 | NS | | | NS | | |

^a^Reported hours per day above IC_50_ are least squares estimates of mixed models as described in the “Statistical analysis” section. Protein binding was accounted for in the calculations.

^b^JAKis were administered once daily (baricitinib and upadacitinib) or twice daily (tofacitinib).

*p<0.01, **p<0.001, ***p<0.0001 compared to baricitinib 4-mg.

bari, baricitinib; G-CSF, granulocyte colony-stimulating factor; GM-CSF, granulocyte-macrophage colony-stimulating factor; IC_50_, half maximum inhibitory concentration; IFN, interferon; IL, interleukin; JAK, Janus kinase; NS, no stimulation; pSTAT, phosphorylated signal transducer and activator of transcription; tofa, tofacitinib; TYK, tyrosine kinase; upa, upadacitinib.

**Table S3. Hours per day above IC_50_ in CD4+ T cells, NK cells, and monocytes: baricitinib 2-mg^a,b^**

|  | **CD4+ T cells** | | | **NK cells** | | | **Monocytes** | | |
| --- | --- | --- | --- | --- | --- | --- | --- | --- | --- |
| **Stimulation/**  **pSTAT** | Bari  2-mg | Upa  15-mg  30-mg | Tofa  5-mg  10-mg | Bari  2-mg | Upa  15-mg  30-mg | Tofa  5-mg  10-mg | Bari  2-mg | Upa  15-mg  30-mg | Tofa  5-mg  10-mg |
| **JAK1/3-dependent cytokines** |  |  |  |  |  |  |  |  |  |
| IL-2/pSTAT5 | 1.9 | 15.3***  20.6*** | 23.8***  24.2*** | 0.2 | 6.6**  10.5*** | 21.4***  24.0*** | NS | | |
| IL-4/pSTAT6 | 0.3 | 9.9*  14.4*** | 19.6***  24.2*** | 4.8 | 17.5***  22.3*** | 24.0***  24.0*** | 0.1 | 8.2**  12.3*** | 12.3***  19.7*** |
| IL-15/pSTAT5 | 0 | 10.4***  14.5*** | 21.2***  24.0*** | 0 | 3.4  8.1** | 17.0***  23.0*** | NS | | |
| IL-21/pSTAT3 | 0 | 9.0***  13.1*** | 17.0***  23.6*** | 0 | 7.7**  11.6*** | 17.5***  23.9*** | 0 | 4.4  8.6** | 12.5*  18.9** |
| **JAK2/2- or JAK2/TYK2-dependent cytokines** |  |  |  |  |  |  |  |  |  |
| IL-3/pSTAT5 | NS | | | NS | | | 2.8 | 13.0***  18.3*** | 0.3*  8.1** |
| G-CSF/pSTAT3 | NS | | | NS | | | 0 | 0.1  3.2 | 2.3  8.5* |
| GM-CSF/pSTAT5 | NS | | | NS | | | 2.5 | 12.6**  18.1*** | 2.2  9.0*** |
| **JAK1/JAK2/TYK2-dependent cytokines** |  |  |  |  |  |  |  |  |  |
| IL-6/pSTAT3 | 0 | 0.6  5.5* | 7.3**  14.8*** | NS | | | 0.2 | 3.1  7.6** | 10.8**  18.2*** |
| IL-10/pSTAT3 | 0 | 0  2.8 | 7.6**  15.1*** | 0 | 0  0.5 | 4.2*  11.8*** | 0 | 0.6  3.5 | 0.8  7.8** |
| IFN-γ/pSTAT1 | NS | | | NS | | | 0.2 | 5.8**  9.7*** | 9.4***  16.8*** |
| IFN-α/pSTAT1 | 0 | 3.6  8.1* | 0  5.2 | 0 | 0  4.8** | 0.1  5.7** | 0 | 2.9  7.5** | 0  2.8*** |
| IFN-α/pSTAT3 | 2.1 | 10.4*  15.0** | 8.0***  15.5*** | NS | | | 9 | 21.7**  23.7** | 16.6**  23.2*** |
| IFN-α/pSTAT5 | 4.4 | 12.9*  17.2* | 12.2***  19.6*** | NS | | | 10.3 | 21.9**  23.5*** | 17.2***  23.9*** |

^a^Reported hours per day above IC_50_ are least squares estimates of mixed models as described in the “Statistical analysis” section. Protein binding was accounted for in the calculations.

^b^JAKis were administered once daily (baricitinib and upadacitinib) or twice daily (tofacitinib).

*p<0.01, **p<0.001, ***p<0.0001 compared to baricitinib 2-mg.

bari, baricitinib; G-CSF, granulocyte colony-stimulating factor; GM-CSF, granulocyte-macrophage colony-stimulating factor; IC_50_, half maximum inhibitory concentration; IFN, interferon; IL, interleukin; JAK, Janus kinase; JAKi, JAK inhibitor; NK, natural killer; NS, no stimulation; pSTAT, phosphorylated signal transducer and activator of transcription; tofa, tofacitinib; TYK, tyrosine kinase; upa, upadacitinib.

**Table S4. Hours per day above IC_50_ in B cells and CD8+ T cells: baricitinib 2-mg^a,b^**

|  | **B cells** | | | **CD8+ T cells** | | |
| --- | --- | --- | --- | --- | --- | --- |
| **Stimulation/**  **pSTAT** | Bari  2-mg | Upa  15-mg  30-mg | Tofa  5-mg  10-mg | Bari  2-mg | Upa  15-mg  30-mg | Tofa  5-mg  10-mg |
| **JAK1/3-dependent cytokines** |  |  |  |  |  |  |
| IL-2/pSTAT5 | NS | | | 3.0 | 12.4***  17.0*** | 22.0***  23.2*** |
| IL-4/pSTAT6 | 0 | 3.3  7.8** | 7.3*  14.8*** | 1.9 | 12.1*  16.7** | 23.5***  24.1*** |
| IL-15/pSTAT5 | NS | | | 0 | 5.7*  9.6*** | 16.2***  23.1*** |
| IL-21/pSTAT3 | 0 | 7.4***  11.3*** | 14.4***  21.8*** | 0 | 7.7***  11.7*** | 16.7***  23.3*** |
| **JAK2/2- or JAK2/TYK2-dependent cytokines** |  |  |  |  |  |  |
| IL-3/pSTAT5 | NS | | | NS | | |
| G-CSF/pSTAT3 | NS | | | NS | | |
| GM-CSF/pSTAT5 | NS | | | NS | | |
| **JAK1/JAK2/TYK2-dependent cytokines** |  |  |  |  |  |  |
| IL-6/pSTAT3 | NS | | | NS | | |
| IL-10/pSTAT3 | NS | | | 0 | 0  1.5 | 4.6*  12.3*** |
| IFN-γ/pSTAT1 | 3.6 | 7.9  12.5 | 14.1***  21.1*** | NS | | |
| IFN-α/pSTAT1 | 0 | 0  3.9*** | 0  1.3 | 0.1 | 0.7  5.7 | 0.1  3.3* |
| IFN-α/pSTAT3 | NS | | | NS | | |
| IFN-α/pSTAT5 | NS | | | NS | | |

^a^Reported hours per day above IC_50_ are least squares estimates of mixed models as described in the “Statistical analysis” section. Protein binding was accounted for in the calculations.

^b^JAKis were administered once daily (baricitinib and upadacitinib) or twice daily (tofacitinib).

*p<0.01, **p<0.001, ***p<0.0001 compared to baricitinib 2-mg.

bari, baricitinib; G-CSF, granulocyte colony-stimulating factor; GM-CSF, granulocyte-macrophage colony-stimulating factor; IC_50_, half maximum inhibitory concentration; IFN, interferon; IL, interleukin; JAK, Janus kinase; JAKi, JAK inhibitor; NS, no stimulation; pSTAT, phosphorylated signal transducer and activator of transcription; tofa, tofacitinib; TYK, tyrosine kinase; upa, upadacitinib.

**Table S5. Average daily percent STAT inhibition in B cells and CD8+ T cells: baricitinib 4-mg^a,b^**

|  | **B cells** | | | **CD8+ T cells** | | |
| --- | --- | --- | --- | --- | --- | --- |
| **Stimulation/**  **pSTAT** | Bari  4-mg | Upa  15-mg  30-mg | Tofa  5-mg  10-mg | Bari  4-mg | Upa  15-mg  30-mg | Tofa  5-mg  10-mg |
| **JAK1/3-dependent cytokines** |  |  |  |  |  |  |
| IL-2/pSTAT5 | NS | | | 48 | 52  64** | 76***  87*** |
| IL-4/pSTAT6 | 11 | 27*  39** | 37**  56*** | 43 | 51  62* | 77***  88*** |
| IL-15/pSTAT5 | NS | | | 24 | 32  45*** | 60***  76*** |
| IL-21/pSTAT3 | 21 | 41***  52*** | 53***  69*** | 25 | 40**  52*** | 60***  76*** |
| **JAK2/2- or JAK2/TYK2- dependent cytokines** |  |  |  |  |  |  |
| IL-3/pSTAT5 | 21 | 22  30 | 10*  17 | NS | | |
| G-CSF/pSTAT3 | NS | | | NS | | |
| GM-CSF/pSTAT5 | NS | | | NS | | |
| **JAK1/JAK2/TYK2-dependent cytokines** |  |  |  |  |  |  |
| IL-6/pSTAT3 | NS | | | NS | | |
| IL-10/pSTAT3 | NS | | | 25 | 17  25 | 34*  47*** |
| IFN-γ/pSTAT1 | 48 | 41  52 | 52  67** | NS | | |
| IFN-α/pSTAT1 | 17 | 15  25* | 15  26** | 23 | 23  34 | 19***  30*** |
| IFN-α/pSTAT3 | NS | | | NS | | |
| IFN-α/pSTAT5 | NS | | | NS | | |

^a^Reported average daily percent STAT inhibition values are least squares estimates of mixed models as described in the “Statistical analysis” section. Protein binding was accounted for in the calculations.

^b^JAKis were administered once daily (baricitinib and upadacitinib) or twice daily (tofacitinib).

*p<0.01, **p<0.001, ***p<0.0001 compared to baricitinib 4-mg.

bari, baricitinib; G-CSF, granulocyte colony-stimulating factor; GM-CSF, granulocyte-macrophage colony-stimulating factor; IFN, interferon; IL, interleukin; JAK, Janus kinase; JAKi, JAK inhibitor; NS, no stimulation; pSTAT, phosphorylated STAT; STAT, signal transducer and activator of transcription; tofa, tofacitinib; TYK, tyrosine kinase; upa, upadacitinib.

**Table S6. Average daily percent STAT inhibition in CD4+ T cells, NK cells, and monocytes: baricitinib 2-mg^a,b^**

|  | **CD4+ T cells** | | | **NK cells** | | | **Monocytes** | | |
| --- | --- | --- | --- | --- | --- | --- | --- | --- | --- |
| **Stimulation/**  **pSTAT** | Bari  2-mg | Upa  15-mg  30-mg | Tofa  5-mg  10-mg | Bari  2-mg | Upa  15-mg  30-mg | Tofa  5-mg  10-mg | Bari  2-mg | Upa  15-mg  30-mg | Tofa  5-mg  10-mg |
| **JAK1/3-dependent cytokines** |  |  |  |  |  |  |  |  |  |
| IL-2/pSTAT5 | 28 | 60***  71*** | 78***  89*** | 19 | 36***  48*** | 72***  85*** | NS | | |
| IL-4/pSTAT6 | 16 | 45***  57*** | 69***  84*** | 34 | 61***  71*** | 82***  91*** | 18 | 40***  52*** | 51***  70*** |
| IL-15/pSTAT5 | 21 | 47***  59*** | 72***  85*** | 12 | 27*  39** | 62***  79*** | NS | | |
| IL-21/pSTAT3 | 15 | 44***  55*** | 61***  76*** | 15 | 40***  52*** | 61***  76*** | 13 | 34*  45** | 46**  63*** |
| **JAK2/2- or JAK2/TYK2-dependent cytokines** |  |  |  |  |  |  |  |  |  |
| IL-3/pSTAT5 | NS | | | NS | | | 30 | 52**  61*** | 27  42* |
| G-CSF/pSTAT3 | NS | | | NS | | | 14 | 17  26* | 27*  40*** |
| GM-CSF/pSTAT5 | NS | | | NS | | | 32 | 55***  65*** | 23***  37** |
| **JAK1/JAK2/TYK2- dependent cytokines** |  |  |  |  |  |  |  |  |  |
| IL-6/pSTAT3 | 17 | 22  33* | 39***  54*** | NS | | | 19 | 27*  39*** | 46***  61*** |
| IL-10/pSTAT3 | 16 | 21  29* | 39**  53*** | 14 | 14  21 | 34***  48*** | 7 | 17*  26** | 25***  40*** |
| IFN-γ/pSTAT1 | NS | | | NS | | | 21 | 34**  46*** | 43***  61*** |
| IFN-α/pSTAT1 | 19 | 32*  43*** | 24*  36*** | 14 | 21  31*** | 23***  37*** | 12 | 28**  40*** | 21***  32*** |
| IFN-α/pSTAT3 | 30 | 48*  61** | 40*  55*** | NS | | | 44 | 72**  81*** | 60**  76*** |
| IFN-α/pSTAT5 | 33 | 52*  64** | 49***  64*** | NS | | | 46 | 75**  83*** | 62***  78*** |

^a^Reported average daily percent STAT inhibition values are least squares estimates of mixed models as described in the “Statistical analysis” section. Protein binding was accounted for in the calculations.

^b^JAKis were administered once daily (baricitinib and upadacitinib) or twice daily (tofacitinib).

*p<0.01, **p<0.001, ***p<0.0001 compared to baricitinib 2-mg.

bari, baricitinib; G-CSF, granulocyte colony-stimulating factor; GM-CSF, granulocyte-macrophage colony-stimulating factor; IFN, interferon; IL, interleukin; JAK, Janus kinase; JAKi, JAK inhibitor; NK, natural killer; NS, no stimulation; pSTAT, phosphorylated STAT; STAT, signal transducer and activator of transcription; tofa, tofacitinib; TYK, tyrosine kinase; upa, upadacitinib.

**Table S7. Average daily percent STAT inhibition in B cells and CD8+ T cells: baricitinib 2-mg^a,b^**

|  | **B cells** | | | | | **CD8+ T cells** | | | |
| --- | --- | --- | --- | --- | --- | --- | --- | --- | --- |
| **Stimulation/**  **pSTAT** | Bari  2-mg | | | Upa  15-mg  30-mg | Tofa  5-mg  10-mg | Bari  2-mg | | Upa  15-mg  30-mg | Tofa  5-mg  10-mg |
| **JAK1/3-dependent cytokines** | | |  |  |  | |  |  |  |
| IL-2/pSTAT5 | NS | | | | | 32 | | 52***  64*** | 76***  87*** |
| IL-4/pSTAT6 | 5 | | | 27*  39*** | 37**  56*** | 26 | | 51*  62*** | 77***  88*** |
| IL-15/pSTAT5 | NS | | | | | 12 | | 32**  45*** | 60***  76*** |
| IL-21/pSTAT3 | 11 | | | 41***  52*** | 53***  69*** | 14 | | 40***  52*** | 60***  76*** |
| **JAK2/2- or JAK2/TYK2-dependent cytokines** | |  | |  |  | |  |  |  |
| IL-3/pSTAT5 | 11 | | | 22  30 | 10  17 | | NS | | |
| G-CSF/pSTAT3 | NS | | | | | | NS | | |
| GM-CSF/pSTAT5 | NS | | | | | | NS | | |
| **JAK1/JAK2/TYK2-dependent cytokines** | |  | |  |  | |  |  |  |
| IL-6/pSTAT3 | NS | | | | | | NS | | |
| IL-10/pSTAT3 | NS | | | | | | 16 | 17  25 | 34***  47*** |
| IFN-γ/pSTAT1 | 33 | | | 41  52 | 52**  67*** | | NS | | |
| IFN-α/pSTAT1 | 8 | | | 15*  25*** | 15*  26*** | | 13 | 23  34** | 19***  30*** |
| IFN-α/pSTAT3 | NS | | | | | | NS | | |
| IFN-α/pSTAT5 | NS | | | | | | NS | | |

^a^Reported average daily percent STAT inhibition values are least squares estimates of mixed models as described in the “Statistical analysis” section. Protein binding was accounted for in the calculations.

^b^JAKis were administered once daily (baricitinib and upadacitinib) or twice daily (tofacitinib).

*p<0.01, **p<0.001, ***p<0.0001 compared to baricitinib 2-mg.

bari, baricitinib; G-CSF, granulocyte colony-stimulating factor; GM-CSF, granulocyte-macrophage colony-stimulating factor; IFN, interferon; IL, interleukin; JAK, Janus kinase; JAKi, JAK inhibitor; NS, no stimulation; pSTAT, phosphorylated STAT; STAT, signal transducer and activator of transcription; tofa, tofacitinib; TYK, tyrosine kinase; upa, upadacitinib.


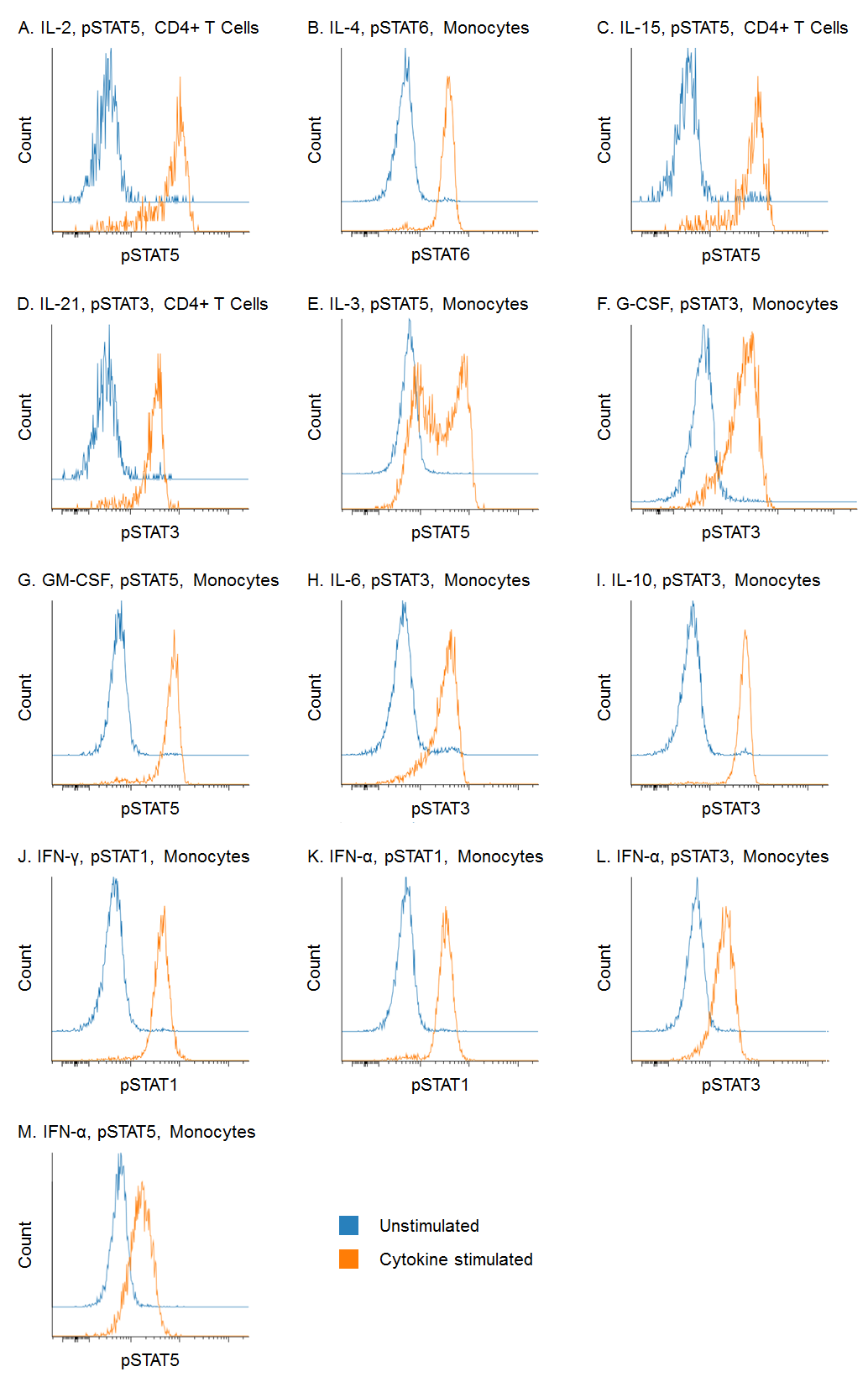


**Figure S1. Select representative histogram plots for cytokine-induced STAT phosphorylation**

Representative histogram plots for cytokine-induced STAT phosphorylation is shown for (A) IL-2/pSTAT5 in CD4+ T cells, (B) IL-4/pSTAT6 in monocytes, (C) IL-15/pSTAT5 in CD4+ T cells, (D) IL-21/pSTAT3 in CD4+ T cells, (E) IL-3/pSTAT5 in monocytes, (F) G-CSF/pSTAT3 in monocytes, (G) GM-CSF/pSTAT5 in monocytes, (H) IL-6/pSTAT3 in monocytes, (I) IL-10/pSTAT3 in monocytes, (J) IFN-γ/pSTAT1 in monocytes, (K) IFN-α/pSTAT1 in monocytes, (L) IFN-α/pSTAT3 in monocytes, (M) IFN-α/pSTAT5 in monocytes. Cells were unstimulated or stimulated with cytokine as described in the Methods. G-CSF, granulocyte colony-stimulating factor; GM-CSF, granulocyte-macrophage colony-stimulating factor; IFN, interferon; IL, interleukin; NK, natural killer; pSTAT, phosphorylated STAT; STAT, signal transducer and activator of transcription.
